# Use of two autochthonous bacteriocinogenic strains as starter cultures in the production of *salchichónes*, a type of Spanish fermented sausages

**DOI:** 10.1016/j.crfs.2023.100615

**Published:** 2023-10-11

**Authors:** J. David García-López, Federica Barbieri, Alberto Baños, Jose Manuel Garcia Madero, Fausto Gardini, Chiara Montanari, Giulia Tabanelli

**Affiliations:** a Department of Microbiology, DOMCA S.A.U, 18620, Alhendín, Spain; b Department of Agricultural and Food Sciences, University of Bologna, 47521, Cesena, Italy; c Interdepartmental Center for Industrial Agri-Food Research, University of Bologna, 47521, Cesena, Italy; d Department of Agricultural and Food Sciences, University of Bologna, 40127, Bologna, Italy

**Keywords:** Autochthonous starter strains, Fermented sausages, Bioprotective cultures, *Listeria monocytogenes*, Biogenic amines

## Abstract

In this work, two autochthonous LAB strains (*Lactiplantibacillus paraplantarum* BPF2 and *Pediococcus acidilactici* ST6), isolated from spontaneously fermented sausages produced in Spain, were tested to produce Spanish fermented sausages (*salchichón*) in pilot plants, due to their promising technological and anti-listerial activity. These products were compared with a sample obtained with a commercial starter (RAP) and a spontaneously fermented control sample. Physico-chemical parameters, microbial counts, metagenomic analysis, biogenic amines content and organoleptic profile of the obtained samples were studied to assess the performances of the native starters. In fact, traditional and artisanal products obtained through spontaneous fermentations can represent an important biodiversity reservoir of strains to be exploited as new potential starter cultures, to improve the safety, quality and local differentiation of traditional products. The data underlined that ST6 strain resulted in a final lower percentage if compared with the other LAB used as starter cultures. The use of starters reduced the BA concentration observed in the sausages obtained with spontaneous fermentation and the BPF2 and ST6 strains were able to decrease the level of products rancidity.

Moreover, a challenge test against *L. monocytogenes* were performed. The data confirmed the effectiveness in the inhibition of *L. monocytogenes* by the two bacteriocinogenic strains tested, with respect to RAP and control samples, highlighting their ability to produce bacteriocins in real food systems. This work demonstrated the promising application in meat industry of these autochthonous strains as starter cultures to improve sensory differentiation and recognizability of typical fermented sausages.

## Highlights

• Native cultures with antimicrobial activity have been tested as meat starters.
• The use of bacteriocinogenic starters affected sausage microbiota composition.
• Autochthonous starters reduced the content of biogenic amines.
• Autochthonous starters improved recognizability of typical fermented sausages.
• Bacteriocinogenic strains inhibited *Listeria monocytogenes* during ripening.

## Graphical abstract

**Figure:**
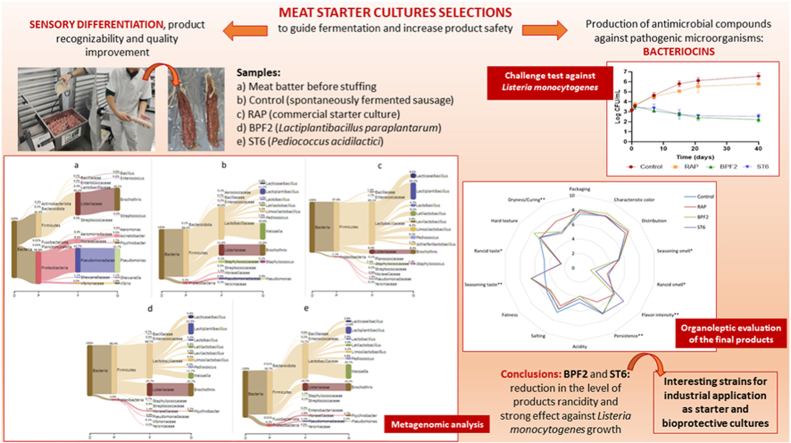

## 1 Introduction

Fermented meat sausages are produced using lean meat and fat added with salt, spices and other additives, such as nitrates, nitrites, ascorbate and sugar. The meat mixture is stuffed in natural or artificial casings and subjected to fermentation and drying process (ripening), which brings to modification in parameters such as pH and a_w_, allowing to obtain a final product with the desired organoleptic and sensorial characteristics and sausage safety. The microbial activity is guided throughout fermentation and ripening by lactic acid bacteria (LAB) and coagulase negative staphylococci (CNS) (in some cases micrococci). Moreover, in Mediterranean tradition, fungi are also used, among which molds and, eventually, yeasts (Laranjo et al., 2019; García-Díez and Saraiva, 2021).

LAB play their primary role in the first days after manufacturing, contributing to lower the pH by lactate production through sugar fermentation, which is relevant to reduce the growth potential of undesired bacteria (spoilers or pathogens) and to reach the isoelectric point of meat protein, thus favoring the water evaporation and textural properties acquisition (Cocconcelli and Fontana, 2010). On the other side, staphylococci contribute to flavor formation and nitrate reduction, while molds are necessary to establish the correct dehydration conditions and concur to flavor formation (Carballo, 2012; Cocconcelli and Fontana, 2010; Berni, 2015).

The first indications concerning the possibility to use selected starter cultures to guide the fermentation of salamis go back to the Fifties of the last centuries (Niinivaara 1955; 1964) and, nowadays, their use is common in industrial productions (Laranjo et al., 2019).

Regarding the selection of LAB starter cultures, technological, safety and quality criteria are considered (Laulund et al., 2017). Among the first, the most important are described by the acidification rate, through the homofermentative pathway, the growth in the presence of high NaCl concentration, the resistance to nitrate and nitrite, good growth performances at the temperature adopted in the manufacturing protocols and the ability to survive and multiply under the nutritional stressing conditions, that characterised fermented sausages during ripening. The safety issues mainly consist in the inability to produce biogenic amines (BA) and the absence of antibiotic-resistance horizontally transmissible (Talon and Leroy, 2011; Cocconcelli and Fontana, 2010; Laranjo et al., 2019). Finally, the main quality parameter considered are the enzymatic activity (lipases and proteases) and their contribution to aroma profile formation (Carballo, 2012).

Recently, another important character is considered to select starter cultures. This consists in the bioprotective antimicrobial activity against pathogenic species, such as *Salmonella* spp., *Listeria monocytogenes*, *Escherichia coli* ETEC or *Staphylococcus aureus*. This bioprotective aspect depends not only on the higher competition of selected cultures expressed in the environment, but also on their possibility to produce specific molecules characterised by bacteriostatic or bactericidal effects, such as bacteriocins, reuterin (3-hydroxypropionaldheyde) and other antimicrobial peptides (Woraprayote et al., 2016; Laranjo et al., 2019; Simons et al., 2020; Pérez-Ramos et al., 2021; Yap et al., 2021). In the last years an increasing trend of outbreaks linked to the consumption of fermented sausages have been signaled (Holck et al., 2017; Omer et al., 2018). This increase is related to several factors, among which the general trend in NaCl reduction, the diminution or elimination of nitrate and nitrite salts and not correct procedures in fermentation and drying processes are the most important.

Despite of the great diffusion of starter cultures in fermented sausage industry, in Europe there are still several traditional and artisanal products obtained through spontaneous fermentations (Barbieri et al., 2021). These productions deserve as an important biodiversity reservoir of strains to be exploited as new potential starter cultures (Franciosa et al., 2021). These autochthonous strains could favor the local differentiation of traditional products, affected by the standardization due to the relative low number of selected cultures available (Van Reckem et al., 2019), and could improve the safety of the products depending on their bioprotective features. In fact, several safety concerns can be related to small-scale traditional productions, including the presence and possible growth of *Listeria monocytogenes*, that is among the most frequently detected pathogens in dry fermented sausages (Drosinos et al., 2006). Given its high tolerance to low pH, high salt concentration, this pathogen is highly difficult to control in fermented sausage processing environments where it can persist over time forming assemblages of surface-associated microbial cells and biofilms (Meloni, 2015).

During the previous studies several LAB strains have been isolated in spontaneously fermented sausages produced in Andalusia (Spain). Among these, two strains were characterised by a promising anti-listerial activity. In fact, the strains *Lactiplantibacillus paraplantarum* BPF2 and *Pediococcus acidilactici* ST6 produced bacteriocins (leucocin K and pediocin PA-1 respectively) active against *L. monocytogenes* (García-López et al., 2023).

In this work, the strains *Lpb. Paraplantarum* BPF2 and *P. acidilactici* ST6 were tested as starter cultures to produce Spanish fermented sausages (*salchichón*) in pilot plants. These strains have been characterised for their safety and technological properties, such as growth at different temperatures (10, 20 and 30 °C) and with different NaCl concentrations (0, 2.5 and 5%) before their use as autochthonous starter cultures. Moreover, a sample added with a commercial starter culture and a control sample (obtained without any starter culture by spontaneous fermentation) were also produced. The sausages fermentation and ripening were monitored by analyzing physico-chemical parameters (pH and weight loss), product microbiota, aroma profiles of the finished products and BA content. Finally, a further production in the same conditions was done to perform a challenge test against *L. monocytogenes*.

## 2 Materials and methods

### 2.1 LAB strains and growth conditions

*Lactiplantibacillus paraplantarum* BPF2 and *P. acidilactici* ST6, were isolated from Spanish spontaneously fermented sausages (García-López et al., 2023). The strains were stored in 20% (w/v) glycerol at −80 °C and pre-cultivated for 24 h at 30 °C in de Man-Rogosa-Sharpe (MRS) broth (Scharlab, Barcelona, Spain) before their characterisation and use.

### 2.2 Safety and technological LAB strains characterisation

Regarding safety aspects, the amino biogenic potential of *Lpb. Paraplantarum* BPF2 and *P. acidilactici* ST6 was tested through the screening in Bover-Cid-Holzapfel medium (BC) according to the method proposed by Bassi et al. (2022). Moreover, the two LAB strains were evaluated for their growth performances in MRS broth at different incubation temperatures (10, 20 and 30 °C) and in relation of different salt concentrations (0, 2.5 and 5% NaCl), starting from a concentration of about 6 log CFU/mL. Samples characterised by different salt concentrations were incubated at 20 °C. During incubation, strains growth was monitored through the variation of optical density at 600 nm (OD_600_), measured with an UV-VIS spectrophotometer 6705 UV–Vis (Jenway, Stone, UK).

### 2.3 *Salchichón* production

The fermented sausages used in these analyses were produced in meat products pilot plant from DOMCA (Granada, Spain). They were prepared with lean pork meat (75%) and pork fat (25%) and a total of 40 kg of meat batter were minced in an 8 mm hole plate grinder (Braher International, Donostia, Spain) and mixed (Mainca, Barcelona, Spain) for 5 min with salt (2.3%), dextrose (0.5%) and a commercial mixture of spices and preservatives, including nitrate (150 mg/kg) and nitrite (30 mg/kg). The mixture was divided into 4 batches (approx. 10 kg each one) into which different LAB cultures were inoculated. The cultures used were *Lpb. Paraplantarum* BPF2, *P. acidilactici* ST6 and a commercial starter culture, containing *Lactiplantibacillus plantarum, Latilactobacillus sakei*, *Staphylococcus xylosus* and *Staphylococcus carnosus* (RAP; Biovitec, Lyon, France). This latter starter culture was employed according to the manufacturer recommendation, reaching a final dosage of approx. 6.3 log CFU/g. The other selected strains were pre-cultivated in MRS broth (Scharlab) for 24 h at 30 °C until reaching a concentration of about 9 log CFU/mL and then, after proper dilutions in sterile saline solution, inoculated in the meat batter at a final concentration similar to those of commercial starter culture (about 6.3 log CFU/g). In addition, a spontaneously fermented control sample, without any LAB culture added, was considered. After that, each batch was split and stuffed separately using a hydraulic stuffer (EC-12, Mainca SL) into natural lamb casings of 50 mm caliber (Villena, Granada, Spain) with a weight of approx. 500 g (20 samples for each batch). Between the stuffing of each batch, a cleaning procedure was carried out with a 1% bleach solution and subsequently rinsed, to avoid cross-contamination with the different microorganisms. The ripening process for each sample was carried out at 18 °C for 10 h, then the samples were maintained at 24 °C for 48 h. Finally, products were dried in a chamber with 80% humidity and a temperature of 14 °C for 28 days.

The obtained samples were monitored and evaluated during all the production process. The analysis was performed in triplicate (three independent sausages for each batch).

### 2.4 Physico-chemical and microbial analyses

Each sample was monitored during ripening regarding physico-chemical parameters and microbiological aspects. In particular, pH was measured by using a pH-meter Basic 20 (Crison Instruments, Barcelona, Spain) during fermentation and ripening processes. Moreover, at each sampling time, sausages were also weighed to calculate the mean weight loss (%) with respect to the initial one. In addition, the principal microbial groups were detected through a microbiological sampling onto selective media. Briefly, 10 g of each sample, obtained by removing aseptically the casing, were transferred into a stomacher bag, mixed with 90 mL of 0.9% (w/v) NaCl sterile solution and homogenized in a Lab Blender Stomacher (Seward Medical, London, UK) for 2 min. Afterwards, appropriate decimal dilutions were prepared and plated onto selective culture media. In particular, LAB, coagulase negative cocci and yeasts were enumerated in MRS Agar added with cycloheximide (200 mg/L), Mannitol Salt Agar (MSA) and Sabouraud Dextrose Agar (SAB) added with chloramphenicol (200 mg/L), respectively, after 48 h of incubation at 30 °C. Moreover, *Enterobacteriaceae* was also detected through Violet Red Bile Glucose Agar (VRBGA), after an incubation of 24 h at 37 °C. All media were provided by Scharlab. All these analyses were performed in triplicate (three different sausages) and the results were expressed as mean value.

### 2.5 Color analysis

The analysis of the color evolution of the sausages was performed by cutting the samples into slices with a thickness of 1 cm, and the readings were taken from the internal surface of the sausages (Seleshe and Kang, 2021). Spectral data for each sample were measured using a PCE-CSM 5 portable colorimeter (PCE Instruments, Meschede, Germany) after calibration with the manufacturer-supplied white calibration plate. For color analysis, an average score for L*, a*, and b × was taken from the mean of three random readings and expressed as L* (lightness), a* (redness), b* (yellowness) using ICD system.

### 2.6 DNA extraction

Total genomic DNA was extracted by taking approximately 2 g of frozen sausage samples, which were treated with lysozyme at 37 °C for 1 h. Subsequently, samples were subjected to a mechanical lysis with glass beads through TissueLyser II (Qiagen, Germantown, USA) with a frequency of 30 Hz for 1 min, followed by proteinase K treatment for 30 min at 60 °C. The DNA was then purified using a DNeasy mericon Food Kit (Qiagen) according to the manufacturer's recommendations. The purified DNA, resuspended in TrisHCl 10 mM, was quantified using a Qubit 4 Fluorimeter (ThermoFisher Scientific, Waltham, USA).

### 2.7 Sequencing and bioinformatic analysis

Libraries were prepared by following Illumina 16 S Metagenomic Sequencing Library Preparation protocol in two amplification steps: an initial 35 cycle PCR amplification using 16 S rDNA V3–V4 specific PCR primers (16 S–341 F 5′- CCTACGGGNBGCASCAG -3′ and 16 S–805 R 5′- GACTACNVGGGTATCTAATCC -3′) and a subsequent amplification that integrates relevant flow-cell binding domains and unique indices (NexteraXT Index Kit, FC-131- 1001/FC-131-1002). Libraries were sequenced on NovaSeq instrument (Illumina, San Diego, USA) using 300 bp paired-end mode. Base calling, demultiplexing and adapter masking were carried out through 10.13039/100010905Illumina BCL Convert v3.9.3 (https://emea.support.illumina.com/). The taxonomic assignment of the filtered and trimmed reads was determined by Kraken 2, which examines the k-mers obtained from sequencing reads with those produced from Silva ribosomal RNA Database (release 138.1) available for Kraken 2 (Wood et al., 2019). Subsequently, Bracken was run on the Kraken2 output files for the estimation of the relative abundance for each taxon identified (Lu et al., 2017), producing reports suitable for visualization with Pavian (Breitwieser and Salzberg, 2020). Data were expressed as relative reads percentage (%) with respect to the total reads generated by the sequencing.

### 2.8 Quantification of biogenic amines content in fermented sausages

BA quantification was determined for each product, collected at the end of the ripening, after an extraction with trichloroacetic acid (TCA) 5%. Briefly, 10 g of sample were treated with 20 mL of TCA at 75 °C for 30 min. After this incubation the solution was filtered into 50 mL flask. This step was repeated twice and at the end the extract acid solution was added until reach a final volume of 50 mL. Samples were then derivatized and injected into HPLC to detect the presence of the principal BA according to the method reported by Montanari et al. (2021). All the analyses were performed in triplicate.

### 2.9 Evaluation of the aroma profile in the finished products

Gas-chromatography-mass spectrometry coupled with the solid-phase microextraction (GC-MS-SPME) technique was employed for the volatile organic compounds (VOCs) analysis of the products at the end of ripening. A known amount of 4-methyl-2-pentanol (Sigma-Aldrich) was added as internal standard to a total of 3 g of samples and analyzed according to the protocol reported by Montanari et al. (2021).

An Agilent Hewlett Packard 7890 GC gas-chromatograph equipped with a MS detector 5975 MSD (Hewlett-Packard) were used to peaks detection, while volatile peaks identification was carried out using Agilent Hewlett-Packard NIST, 2011 mass spectral library (NIST, 2011). The mass spectrum identification was confirmed in the same conditions by injection of the pure standards of the principal compounds (hexanal, acetone, 2-butanone-3-hydroxy, ethyl alcohol, 1-hexanol, acetic acid and hexanoic acid) and the data were expressed as the ratio between each molecules peak area and the peak area of internal standard. Molecules deriving form spices (Montanari et al., 2016) were not considered given the not uniform distribution in the samples. All the analyses were performed in triplicate.

### 2.10 Sensory evaluation of *salchichón*

To simulate consumer evaluations in a more objective and realistic manner, a sensory evaluation was conducted with untrained panelists. A total of 20 individuals, comprising 10 women and 10 men aged between 20 and 60 years, were randomly selected. Samples of sausages from the different treatments were sliced with a thickness of 5 mm, and brought to room temperature before evaluation (Yuan et al., 2021). Panelists were provided with water and unsalted toast to cleanse their palates at the beginning of the session and between samples. Additionally, coffee beans were given to neutralize odors (Cittadini et al., 2022). The scoring of each sample was made according to a 14 points scale to assess consumer preferences for each attribute. On the decimal scale, ‘10′ corresponds to ‘like very much’ and ‘1′ corresponds to ‘dislike very much’. Specifically, the sensory characteristics included: Appearance attributes (color, fat/lean cohesion), odor attributes (rancid), flavor attributes (flavor intensity, persistence, acidity, salty, fatty and rancid) and texture attributes (dryness and texture). All evaluations were determined in the sensory evaluation laboratory, equipped with individual cabinets and white lighting.

### 2.11 Challenge test against *Listeria monocytogenes*

After the characterisation of the fermented sausages obtained through the application of different starter cultures, a challenge test against *L. monocytogenes* DSM 112142 was performed, to evaluate the anti-listerial activity of each tested strain. For this purpose, a new batch of fermented sausages was prepared following the same methodology described in the Paragraph 2.3. The antimicrobial effect was assessed through the inoculum of the target microorganism into the meat batter before stuffing. The pathogen was pre-cultivated in BHI broth medium (Scharlab) at 37 °C for 24 h until reaching a concentration of about 9 log CFU/mL and, after proper dilutions in sterile saline solution, inoculated in the meat batter at a final concentration of about 3.2 log CFU/g. *L. monocytogenes* growth behavior was monitored over time by sampling onto COMPASS Listeria Agar plates (Bioser, Barcelona, Spain). Data were collected after 48 h of incubation at 37 °C.

### 2.12 Statistical analyses

Data collected were statistically analyzed through one-way ANOVA procedure of Statistica 8.0 software (StatSoft Inc., Tulsa, USA) and significant differences of each sample at each time of analysis were evaluated with the LDS test (*p* < 0.05) and highlighted with different letters in the tables. The heat map was obtained in the statistical through statistical software R (R Core Team, 2020).

## 3 Results and Discussion

### 3.1 Strains characterisation regarding safety and technological aspects

The strains *Lpb. Paraplantarum* BPF2 and *P. acidilactici* ST6, chosen for their ability to produce bacteriocins (García-López et al., 2023), were also tested regarding some relevant safety and technological features. In particular, none of them were able to produce BA *in vitro*, according to the screening in Bover-Cid-Holzapfel medium (Bover-Cid and Holzapfel, 1999). Moreover, all the strains were able to grow at 10 and 30 °C and in the presence of salt concentration up to 5%.

### 3.2 Physico-chemical and microbial characterisation of Spanish fermented sausages

Three batches of *salchichón* characterised by different starter cultures and a product obtained through a spontaneous fermentation were monitored over time for controlling the pH dynamics and the weight losses (Table 1 and Table 2, respectively). Results concerning pH showed a marked decrease after 7 days (approx. 0.8 unit). In general, the sample RAP showed significantly lower pH values after the fermentation, followed by the sausages inoculated with BPF2. The samples in which *P. acidilactici* was used as starter culture behaved similarly to the control, showing a lower pH decrease. At the end of ripening, no significant difference was observed between the samples RAP and BPF2 while the other sausages presented a higher pH value. Concerning weight losses, the control was characterised by the slower kinetics, reaching the lowest a final value of 24.61%, compared with decreases observed in fermented sausages with starter cultures (from 28.88% to 31.07%).

**Table 1:** Samples pH variation during the fermentation and ripening.

pH	0 days	7 days	15 days	22 days	30 days
**Control**	6.34 ± 0.03^a^^a^^b^	5.63 ± 0.03^a^	5.44 ± 0.02^a^	5.33 ± 0.03^a^	5.13 ± 0.02^a^
**RAP**	6.29 ± 0.05^a^	5.46 ± 0.04^b^	5.39 ± 0.01^b^	5.20 ± 0.04^b^	5.03 ± 0.03^b^
**BPF2**	6.32 ± 0.03^a^	5.57 ± 0.03^c^	5.42 ± 0.06^a^	5.18 ± 0.02^b^	5.08 ± 0.02^b^
**ST6**	6.37 ± 0.04^a^	5.70 ± 0.06^a^	5.51 ± 0.05^c^	5.35 ± 0.04^a^	5.15 ± 0.04^a^

a Standard deviation.

b Different letters highlight significant differences of each sample at each time of analysis, as a result of ANOVA test (*p* < 0.05).

**Table 2:** Samples weight losses trend during the fermentation and ripening.

Weight loss (%)	0 days	7 days	15 days	22 days	30 days
**Control**	0.00	6.49 ± 0.64^a^^a^^b^	11.55 ± 1.12^a^	18.73 ± 0.21^a^	24.61 ± 0.89^a^
**RAP**	0.00	7.38 ± 0.33^b^	21.13 ± 0.76^b^	23.88 ± 2.54^b^	28.88 ± 2.35^b^
**BPF2**	0.00	6.74 ± 0.41^a^	19.37 ± 2.47^bc^	22.74 ± 2.83^b^	31.07 ± 2.92^b^
**ST6**	0.00	9.33 ± 1.23^c^	17.75 ± 2.83^c^	21.75 ± 1.82^b^	29.06 ± 2.91^b^

a Standard deviation.

b Different letters highlight significant differences of each sample at each time of analysis, as a result of ANOVA test (*p* < 0.05).

Regarding microbial characterisation, the results are shown in Table 3. In the control without starter cultures, the initial LAB count on MRS was 3.30 log CFU/g. This number gradually increased during ripening. However, the final concentration of this group did not exceed a final value of 6.30 log CFU/g. The addition of LAB starter cultures determined initial LAB concentration comprised between 6.24 and 6.41 log CFU/g. After 7 days this microbial group increased up to 8 log CFU/g and more in all the sample and reached, after 30 days, counts higher than 9 log CFU/g independently on the cultures added. Coagulase positive cocci were never detected on MSA. Coagulase negative cocci were present at a concentration of 3.70 log CFU/g and only in the samples RAP, added with a commercial starter culture containing *Staph. Xylosus* and *Staph. Carnosus*, the initial value was 6.62 log CFU/g. In this latter case, the concentration of this microbial group was higher than 8 log CFU/g at the end of ripening. In the other samples they increased up to values ranging from 5.11 to 5.30 log CFU/g, without significant difference. Concerning *Enterobacteriaceae*, the initial contamination (3.58 log CFU/g) did not show relevant increases during ripening, with final values ranging from 3.83 to 3.88 log CFU/g, without differences in relation to the different starter cultures used. Finally, yeasts, characterised by an initial value of 2.30 log CFU/g, increased in all the samples up to 6 log CFU/g and more, but the higher counts were attained in the control samples and in the sausages inoculated with RAP. These high counts of yeasts at the end of ripening were consistent with other similar Spanish fermented sausages (Flores et al., 2015).

**Table 3:** Microbial group concentration (log CFU/g) during the fermentation and ripening of the different fermented sausages.

Microbial groups (Medium)	Time (days)	Control	RAP	BPF2	ST6
**Lactobacilli (MRS)**	**0**	3.30 ± 0.15^a^^a^^b^	6.41 ± 0.09^b^	6.29 ± 0.07^b^	6.24 ± 0.12^b^
	**1**	3.40 ± 0.14^a^	7.28 ± 0.10^b^	7.32 ± 0.09^b^	7.59 ± 0.08^b^
	**7**	4.53 ± 0.07^a^	8.15 ± 0.07^bc^	8.28 ± 0.10^c^	7.93 ± 0.07^b^
	**15**	5.80 ± 0.10^a^	8.22 ± 0.08^b^	8.60 ± 0.07^c^	8.09 ± 0.08^b^
	**22**	5.92 ± 0.07^a^	8.84 ± 0.07^b^	8.37 ± 0.08^c^	8.05 ± 0.09^d^
	**30**	6.29 ± 0.09^a^	9.39 ± 0.07^b^	9.30 ± 0.07^b^	9.05 ± 0.07^c^
**Coagulase-negative cocci (MSA)**	**0**	3.70 ± 0.09^a^	6.62 ± 0.07^b^	3.67 ± 0.13^a^	3.70 ± 0.12^a^
	**1**	3.70 ± 0.08^a^	6.72 ± 0.09^b^	3.90 ± 0.14^c^	3.80 ± 0.11^c^
	**7**	3.99 ± 0.10^a^	6.93 ± 0.09^b^	3.95 ± 0.15^a^	3.95 ± 0.13^a^
	**15**	4.42 ± 0.11^a^	7.20 ± 0.07^b^	4.71 ± 0.08^a^	4.68 ± 0.08^a^
	**22**	4.90 ± 0.07^a^	7.91 ± 0.07^b^	4.95 ± 0.09^a^	4.93 ± 0.07^a^
	**30**	5.11 ± 0.08^a^	8.45 ± 0.08^b^	5.30 ± 0.12^c^	5.22 ± 0.09^c^
**Enterobacteria (VRBGA)**	**0**	3.58 ± 0.20	3.49 ± 0.12	3.52 ± 0.19	3.61 ± 0.16
	**1**	3.67 ± 0.18	3.66 ± 0.11	3.63 ± 0.17	3.68 ± 0.13
	**7**	3.82 ± 0.16	3.70 ± 0.16	3.79 ± 0.10	3.75 ± 0.16
	**15**	3.85 ± 0.10	3.82 ± 0.10	3.85 ± 0.20	3.83 ± 0.14
	**22**	3.86 ± 0.10	3.84 ± 0.11	3.87 ± 0.11	3.83 ± 0.14
	**30**	3.87 ± 0.12	3.85 ± 0.11	3.88 ± 0.12	3.83 ± 0.13
**Yeasts (SAB)**	**0**	2.30 ± 0.10	2.32 ± 0.17	2.21 ± 0.20	2.34 ± 0.18
	**1**	2.49 ± 0.18	2.52 ± 0.14	2.53 ± 0.16	2.55 ± 0.16
	**7**	4.54 ± 0.10	4.55 ± 0.01	4.47 ± 0.12	4.37 ± 0.14
	**15**	5.90 ± 0.14^a^	5.67 ± 0.09^ab^	5.53 ± 0.13^b^	5.17 ± 0.14^c^
	**22**	5.91 ± 0.11^a^	5.70 ± 0.10^b^	5.64 ± 0.12^b^	5.48 ± 0.13^c^
	**30**	6.77 ± 0.07^a^	6.81 ± 0.07^a^	6.37 ± 0.07^b^	6.01 ± 0.09^c^

a Standard deviation.

b Different letters highlight significant differences of each sample at each time of analysis, as a result of ANOVA test (*p* < 0.05).

### 3.3 Color analysis

Color is an important attribute in sausages and can affect their overall quality (Hu et al., 2008). Table 4 presents the instrumental color results during a 30-day maturation period for control sausages and sausages inoculated with starter cultures: RAP, BPF2, or ST6. The results indicate that, after the maturation period, the L* parameter showed significantly lower values (*p* > 0.05) in samples inoculated with the starter cultures BPF 2 and ST6 when compared to the other samples. This fact could suggest a potential reduction of dark color, possibly attributed to water loss (Sanabria et al., 2004). The observed decrease in redness values a × may be attributed to signs of oxidation during the maturation process as indicated by Ergezer et al. (2018). Similarly, the decrease in b × values could be attributed to the development of yellowing resulting from reactions between lipid oxidation products and amines (Liu et al., 2019). However, no significant differences were found during the latter half of the curing process for the a × and b × values. Ultimately, at the end of the process, no significant differences were observed for any color parameter between the control and treatments.

**Table 4:** Evaluation of color parameters (L^b^, a^b^ and b^b^) during the fermentation and ripening of the fermented sausages.

Parameter	Time (days)	Control	RAP	BPF2	ST6
**L**^b^	**0**	35.34 ± 0.87^a^	35.34 ± 0.87	35.34 ± 0.87	35.34 ± 0.87
	**1**	31.53 ± 1.92	34.15 ± 2.34	30.05 ± 0.98	32.58 ± 3.01
	**7**	24.78 ± 2.03^a^^b^	28.07 ± 3.73^a^	35.07 ± 4.03^b^	34.57 ± 2.27^b^
	**15**	30.72 ± 1.08^a^	29.76 ± 1.37^a^	26.40 ± 0.13^ab^	23.46 ± 0.88^b^
	**22**	17.79 ± 0.97^a^	23.50 ± 1.02^b^	23.19 ± 0.90^b^	23.12 ± 0.53^b^
	**30**	19.20 ± 0.85^a^	17.59 ± 0.48^a^	21.79 ± 1.47^ab^	23.78 ± 0.48^b^
**a**^b^	**0**	18.23 ± 0.98	18.23 ± 0.98	18.23 ± 0.98	18.23 ± 0.98
	**1**	13.44 ± 0.75^a^	16.38 ± 0.10^b^	17.33 ± 1.31^b^	16.09 ± 0.70^b^
	**7**	12.77 ± 0.65^a^	14.59 ± 0.85^b^	16.48 ± 1.44^b^	16.73 ± 1.13^b^
	**15**	15.43 ± 1.75^a^	15.77 ± 1.01^a^	12.37 ± 0.66^b^	8.83 ± 0.43^c^
	**22**	8.87 ± 0.41	9.13 ± 0.74	9.91 ± 0.34	10.77 ± 0.65
	**30**	10.26 ± 0.13	9.15 ± 0.34	8.77 ± 0.42	8.79 ± 0.70
**b**^b^	**0**	8.61 ± 0.40^b^	8.61 ± 0.40	8.61 ± 0.40	8.61 ± 0.40
	**1**	8.13 ± 0.68	8.39 ± 0.67	7.84 ± 1.34	7.88 ± 1.75
	**7**	7.10 ± 0.88^a^	5.96 ± 1.88^a^	7.54 ± 1.11^ab^	8.93 ± 0.58^b^
	**15**	5.21 ± 0.33	5.58 ± 0.69	5.75 ± 0.47	4.51 ± 0.23
	**22**	3.41 ± 0.24	3.39 ± 0.27	3.91 ± 0.17	4.18 ± 0.47
	**30**	2.99 ± 0.10	3.25 ± 0.30	3.16 ± 0.26	3.01 ± 0.46

a Standard deviation.

b Different letters highlight significant differences of each sample at each time of analysis, as a result of ANOVA test (*p* < 0.05).

### 3.4 Metagenomic analysis

**Fig. 1:**
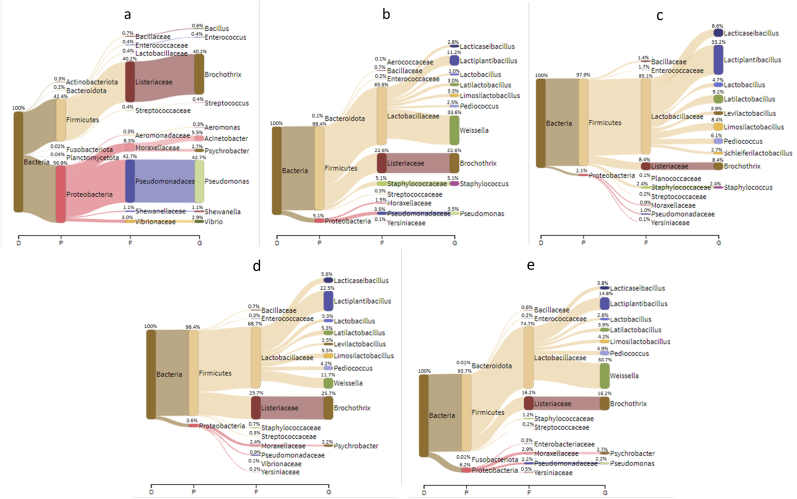
Prevalence (%) of the different genera detected in the *salchichónes* according to metagenomic analysis: a) initial meat batter, b) sample spontaneously fermented at the end of ripening, c) sample with the commercial starter culture RAP, d) sample with *Lpb. Paraplantarum* BPF2, e) sample with *P. acidilactici* SCT6.

A metagenomic analysis was carried out for each sample at the end of ripening in order to highlight the microbial population involved in the product manufacture. In addition, this analysis interested the microbiota of meat batter before starter cultures addition (Fig. 1).

The results concerning the initial meat batter are reported in Fig. 1a. Gram-negative Proteobacteria represented the 56.9% of the reads and, among them, *Pseudomonas* was the most relevant Taxon (42.7%), followed by *Acinetobacter* (5.5%), *Vibrio* (2.9%) and *Psychrobacter* (2.7%). Among Firmicutes (42.4%), *Brochothrix* was the dominant Genus (40.2%). Only sporadic presence of other Gram-positive bacteria was observed, including LAB. Both *Pseudomonas* and *Brochothrix* can not grow in the cultural media used for microbiological analyses, and the quantitative presence of reads of these Genus suggests a relevant initial contamination of the raw material.

In the sausages spontaneously fermented at the end of ripening (Fig. 1b), the presence of Proteobacteria was strongly reduced (5.1% of the reads), with *Pseudomonas* as main Genus (3.5%). Among Firmicutes, *Brochothrix* had still a relevant presence (22.6%), but *Lactobacillaceae* were dominant. In particular, the obligate heterofermentative Genus *Weissella* was the most represented (33.6%). Among lactobacilli, the most important Genus were *Lactiplantibacillus* (11.2%) and *Limosilactobacillus* (3.3%). The presence of *Staphylococcus* was rather limited (5.1%).

In the sausages produced with the commercial starter culture RAP, Proteobacteria (2.1%) and, consequently, *Pseudomonas* (1.0%) were strongly inhibited (Fig. 1c). Also, the presence of *Brochothrix* was extremely limited (8.4%). *Lactobacillaceae* were the most relevant Family and *Lactiplantibacillus* (33.2%) and *Latilactibacillus* (9.2%), added as starter culture, were the dominant Genera. Nevertheless, other LAB species were present in consistent amount, *i.e. Limosilactobacillus* (8.4%, already detected in the not inoculated sausage) and *Lacticaseibacillus* (8.6%). However, *Weissella*, dominant in the control without starter cultures, was non detected in appreciable amounts. The presence of *Staphylococcus* was lower than 2.5%.

The sample in which the starter *Lpb. Paraplantarum* BPF2 (Fig. 1d) has been used showed again a low proportion of Proteobacteria (3.6% of the total reads). Among Firmicutes, the total reads relative to *Brochothrix* (25.7%) were higher, if compared with RAP. Concerning LAB, *Lactiplantibacillus* prevailed (22.5%). However, *Weissella* reached an important proportion (11.7%) together with *Lacticaseibacillus* (5.6%), *Limosilactobacillus* (5.5%) and *Latilactobacillus* (5.3%). Also in this case, the presence of *Staphylococcus* was negligible.

In the sausage obtained by using *P. acidilactici* ST6 (Fig. 1e), the final amount of *Pediococcus* was not high (4.9%) indicating the difficulty of this Genus to grow under the conditions characterising the manufacture of this kind of sausages. On the other side, *Brochothrix* accounted for 16.2% of the total reads. Among LAB, *Weissella* prevailed (30.7%), followed by *Lactiplantibacillus* (14.8%), *Limosilactobacillus* (4.2%), *Latilactobacillus* (3.9%) and *Lacticaseibacillus* (3.8%). Proteobacteria accounted for 6.2% of total reads (*Pseudomonas* 2.2% and *Psychrobacter* 2.7%).

**Fig. 2:**
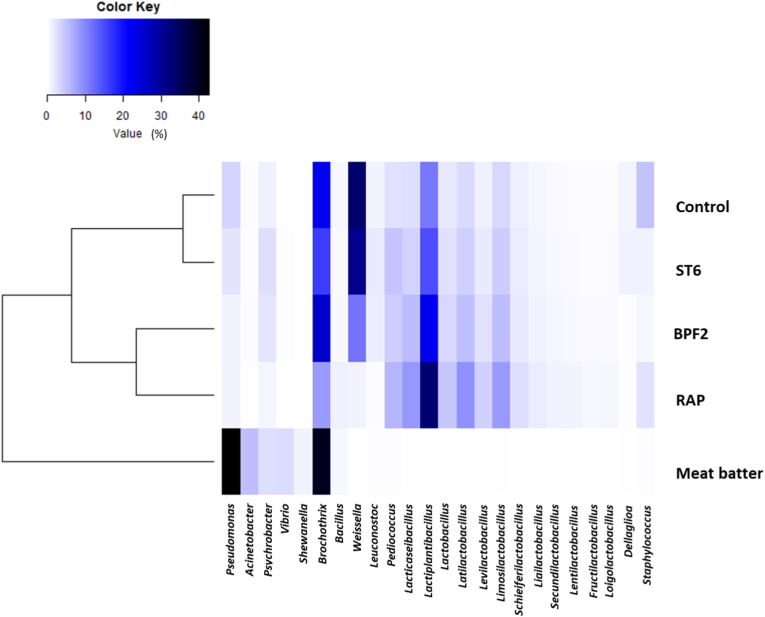
Heatmap relative to the composition of samples microbiota.

The composition of the microbiota of meat batter is summarized in the heat map reported in Fig. 2 in relation to the prevalence of the different genera in the samples. As expected, meat batter had a peculiar profile. The sausages obtained using RAP and BPF2 presented a similar microbiota composition, while the samples obtained with ST6 were closer to the spontaneously fermented Control.

According to the data reported, Gram-negative bacteria, and particularly *Pseudomonas*, were the dominant microorganisms in the meat batter. However, the condition applied (mainly NaCl addition) inhibited, as expected, their growth, especially in the sample containing the commercial started RAP. On the other side, it is well known that *Brochothrix thermosphacta*, a facultatively anaerobic bacteria belonging to *Listeriaceae*, is a typical spoiler of meat product, also due to its psychotropic aptitude, including dry fermented sausages (Li et al., 2021; Xu et al., 2022; Dučić et al., 2023). A similar composition of microbial population in meat batter was described by Juárez-Castelán et al. (2019) and Quijada et al. (2018). At the end of ripening its prevalence was strongly reduced only in the sausages produced with RAP.

In general, the addition of LAB starter cultures determined the prevalence of the respective genus in the ripened products, with the exception of *P. acidilactici*. Interestingly, *Lactiplantibacillus* was dominant among lactobacilli also in the sausages spontaneously fermented. Nevertheless, a great variety of lactobacilli genera were found in all samples.

The presence of the heterofermentative LAB *Weissella* in fermented sausage has been already described, even if this Genus was never dominant among LAB in European product (Tremonte et al., 2017; Belleggia et al., 2022a, 2022b; Hu et al., 2022). However, *Weissella hellenica* was signaled as dominant in Chinese sausages (Hu et al., 2020; Wang et al., 2021). In this case, *Weissella* was dominant in spontaneously fermented sausages, but also in the ST6 samples. Only the commercial starter culture RAP was able to prevent its growth.

### 3.5 Detection of biogenic amines content in fermented sausages

Each sample was analyzed also regarding the BA content at the end of ripening by using HPLC technique. Data reported in Table 5 showed that histamine was always under the detection limit in all samples and 2-phenylethylamine was also never detected. However, relevant differences were observed for other amines.

**Table 5:** Biogenic amine detection (mg/kg) in the different samples at the end of ripening.

Samples	Histamine	Tyramine	Putrescine	Cadaverine	Tot
**Control**	-a	150.92 ± 12.10^b^^a^^c^	143.48 ± 10.63^a^	245.06 ± 31.40^a^	**539.46**^**a**^
**RAP**	–	122.37 ± 9.89^b^	8.31 ± 1.29^b^	58.51 ± 3.57^b^	**189.20**^**b**^
**BPF2**	–	93.10 ± 5.68^c^	25.28 ± 2.29^c^	101.45 ± 12.99^c^	**219.84**^**c**^
**ST6**	–	117.85 ± 10.86^b^	19.98 ± 2.23^c^	114.65 ± 11.12^c^	**252.48**^**d**^

a Under the detection limits (3 mg/kg).

b Standard deviation.

c Different letters highlight significant differences in the content of BA in each sample, as a result of ANOVA test (*p* < 0.05).

In particular, tyramine reached its maximum concentration (150.9 mg/kg) in the control, obtained without the addition of starter cultures. A reduced concentration of this BA was detected in samples with the commercial starter RAP and *P. acidilactici* ST6 (approx. 120 mg/kg), while the presence of BPF2 determined the lower accumulation of tyramine. Cadaverine, detected at a concentration of 245.1 mg/kg in the spontaneously fermented samples, was more than halved in the fermented sausages containing ST6 and BPF2 (with concentrations ranging from 101.5 to 114.7 mg/kg), while RAP further reduced this level, reaching a value of 58.5 mg/kg. The most relevant effect concerned putrescine. In fact, this BA was detected at 143.5 mg/kg in the control, while in the other sausages its level was very low, ranging from 8.3 mg/kg (RAP) and 25.3 mg/kg (BPF2).

The total BA content was more than double in sausages produced without starter culture (approx. 540 mg/kg), confirming the positive effect that starter cultures can exert on the accumulation of these compounds (Barbieri et al., 2019; Laranjo et al., 2019). The most relevant effects interested putrescine and cadaverine. Putrescine derives from the decarboxylation of ornithine, which can be produced both by Gram-negative and Gram-positive bacteria through different pathways. In any case, arginine plays a crucial role in the synthesis of this BA (Wunderlichová et al., 2014). The production of cadaverine is often attributed to Gram-negative bacteria. However, the presence of lysine decarboxylase activity has been demonstrated in some lactobacilli (Pessione et al., 2005; Romano et al., 2013; Berthoud et al., 2022), explaining the high presence of this BA in fermented sausages, even if characterised by a low concentration of *Enterobacteriaceae*.

### 3.6 Aroma profile of finished *salchichónes*

**Fig. 3:**
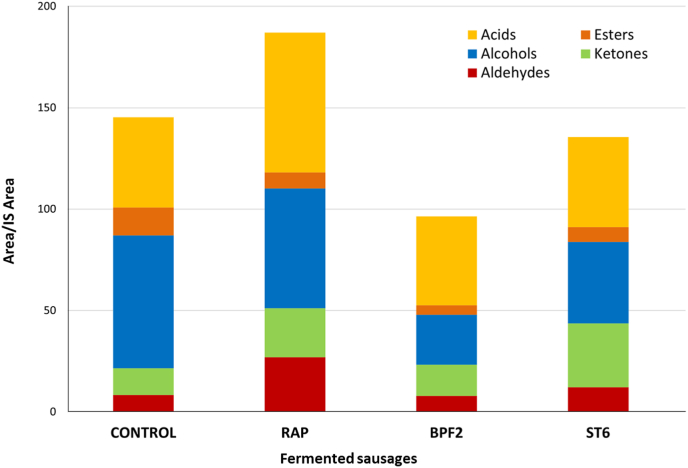
Presence of the different classes of volatile organic compounds (VOCs) in the samples. The values are expressed as the ratio between the peak area of the compound considered and the area of the internal standard.

The volatile profile of the different fermented sausages at the end of ripening is reported in Table 6, in which the molecules detected are grouped according to their chemical structure. Data reported are the mean of 3 different repetition for each sample, which standard deviation was always below 5%. The total amount of each chemical class is showed in Fig. 3.

**Table 6:** Volatile organic compounds (VOCs) of each sample at the end of ripening.

Volatile compounds	Control	RAP	BPF2	ST6
Butanal	-a	0.18^b^	–	0.40
Hexanal	10.12	19.73	3.32	4.69
Nonanal	1.43	2.41	2.12	1.40
Decanal	0.19	0.35	0.30	0.88
Benzaldehyde	0.70	2.22	0.74	1.34
Benzene acetaldehyde	2.24	1.54	0.94	2.73
**Aldehydes**	**14.69**	**26.44**	**7.42**	**11.45**
Acetone	0.80	9.81	5.66	11.68
2-butanone	0.32	0.76	0.52	0.94
2,3-butanedione	–	0.32	–	1.44
2-pentanone	3.61	2.38	1.17	1.40
Methyl isobutyl ketone	0.38	2.22	1.64	3.02
3-hexen-2-one	2.68	3.78	2.71	2.50
2-octanone	1.30	0.97	0.45	0.56
2-butanone-3-hydroxy	2.20	2.25	2.99	9.43
2-nonanone	1.93	1.68	0.24	0.54
**Ketones**	**12.42**	**14.36**	**9.73**	**19.83**
Ethyl alcohol	28.41	4.90	8.27	19.81
1-pentanol	3.42	4.39	1.40	1.36
2-heptanol	0.68	0.15	–	0.55
1-hexanol	22.00	38.86	8.27	4.96
1-octen-3-ol	1.17	3.94	0.87	0.81
1-hexanol, 2-ethyl	1.03	1.42	1.20	3.23
1-octanol	3.02	1.83	0.85	3.00
Nonanol	1.19	–	0.24	0.89
Benzyl alcohol	3.06	2.80	2.33	3.33
Phenylethyl alcohol	1.36	0.73	1.09	2.09
**Alcohols**	**65.36**	**59.03**	**24.53**	**40.02**
Ethyl acetate	4.03	0.80	2.53	2.64
Butanoic acid, ethyl ester	0.88	–	0.18	0.57
Hexanoic acid, ethyl ester	6.83	4.98	1.25	2.32
Acetic acid, hexyl ester	0.32	0.77	–	–
Octanoic acid, ethyl ester	0.97	–	–	0.55
Decanoic acid, ethyl ester	0.42	0.47	0.38	0.73
**Esters**	**13.44**	**7.02**	**4.34**	**6.81**
Acetic acid	28.31	41.11	32.10	30.83
Propanoic acid	0.00	1.40	0.62	0.86
Butanoic acid, 3-methyl	0.98	1.26	1.24	2.20
Pentanoic acid	2.34	2.74	1.03	1.66
Pentanoic acid, 4-methyl	1.09	1.90	0.85	0.52
Hexanoic acid	9.89	10.87	5.52	4.06
Heptanoic acid	0.74	1.11	0.64	0.44
Octanoic acid	–	2.58	–	1.60
Nonanoic acid	1.13	0.73	0.70	0.86
n-decanoic acid	–	1.35	1.13	1.37
**Acids**	**44.47**	**65.05**	**43.84**	**44.39**

a Not detected under the adopted conditions.

b Data are expressed as ratio between peak area of each molecule and peak area of the internal standard (4-methyl-2-pentanol).

Aldehydes were present at higher level in the control and in sausages obtained with the commercial starter culture (RAP). The difference was mainly linked to hexanal (10.12 and 19.73, respectively). This aliphatic aldehyde is the main product of fatty acid autoxidation and derived from linoleic acid (Ordóñez et al., 1999).

Among ketones, acetone, detected in negligible amount in the control, was present at high level in the samples RAP and BPF2, while its presence was more relevant in the samples ST6. Diacetyl (2,3-butanedione) reached its maximum values in the sample ST6, and it is not surprising that acetoin (3-hydroxy-2-butanone), which derive from diacetyl reduction, was higher in the same sample. All these molecules derive from pyruvate metabolisms and indicate different activations of the pathways involving this organic acid (Carballo, 2012; Barbieri et al., 2020, 2022). In addition, 2-pentanone and 2-nonanone were more abundant in the control and in RAP, indicating the possible involvement of yeasts, whose concentration was higher in these samples, in the production of these methyl ketones through β-oxidation of fatty acids.

Ethanol was detected in highest amount in the control, but also the sample ST6 was characterised by high concentration of this alcohol, which showed a low level in RAP. Interestingly, ethanol is one of the end products of pyruvate pathways (Axelsson, 2004). Among alcohols, hexanol, deriving from hexanal reduction, confirmed the previous observations concerning aldehydes: the use of the new selected strains (ST6 and BPF2) allowed the reduction of the presence of products resulting from lipid autoxidation. Similar results were observed for other aliphatic alcohols, such as pentanol and 1-octen-3-ol.

Acetic acid presence resulted higher in the RAP sample, while no relevant differences were observed among the other sausages. Hexanoic (caproic) acid was present at higher proportion in the control and in RAP samples, if compared with the selected strains. Similar results were observed also for pentanoic acid and 4-methyl-pentanoic acid. The origin of hexanoic acid may be attributed to the activity of yeasts, that reached their maximum concentration in these two samples. In fact, it is well known that yeasts can produce short or medium chain fatty acids (Gajewski et al., 2017; Hu et al., 2018). Under these conditions, it is not surprising that ethyl hexanoate (caproate) was the major ester produced in these two samples (control and RAP), while ethyl acetate prevailed in the other fermented sausages.

### 3.7 Sensory evaluation of the products

**Fig. 4:**
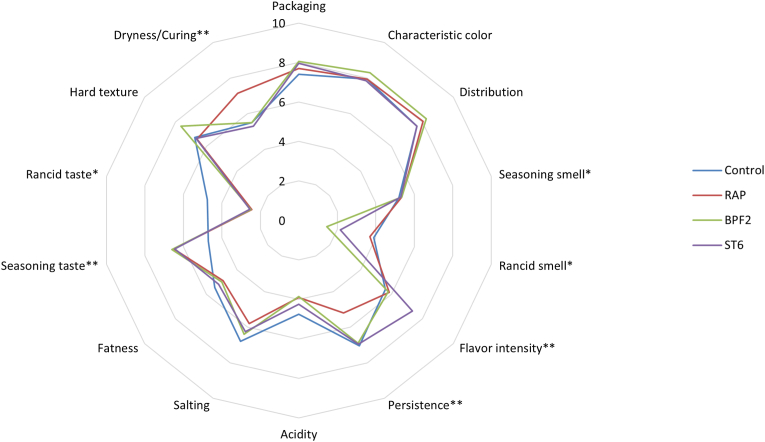
Spider plot of descriptive analysis attributes for sausage samples. The data characterised by significant difference are marked with * (*p* < 0.05) or ** (*p* < 0.01).

Once the maturation process was completed, sensory evaluations were conducted on the sausages. Thus, untrained panelists evaluated 14 parameters of the sausages using a scale from 0 to 10. Fig. 4 shows the results obtained in the different categories. No significant differences were observed among the samples in terms of appearance features, such as packaging, characteristic color, and distribution. With regard to the odor properties, seasoning and rancid odor, panelists found significant differences in rancid aroma for Control and RAP sausages, confirmed by the high content of aldehydes, and in particular hexanal, found in these samples (Table 6). In the case of flavor intensity, this characteristic was significantly higher (*p* < 0.01) in ST6 sausages with respect to the other samples. Significant differences (*p* < 0.05) were also obtained in terms of flavor persistence for ST6, BPF2 and Control samples. No differences were observed between the samples for the taste properties (acidity, salty and fatty). Regarding the textural properties, the RAP sample exhibited greater dryness compared to the other samples. It is important to highlight that this particular type of food displays significant cultural and gastronomic diversity, with its characteristics varying across various regions. It is important to highlight that the assessment of this food category by consumers is heavily influenced by the unique cultural and gastronomic diversity found in each region. In our case, the *salchichones* were produced following traditional artisanal methods typical of Andalusia, which likely positively influenced the evaluation carried out by the panelists. The results obtained showed an improvement in parameters related to positive characteristics of the food produced with BPF2 and ST6 strains, such as the intensity and persistence of its characteristic flavor (Denktas et al., 2021). Furthermore, these *salchichones* exhibit a reduced level of taste and aroma rancidity, which is considered undesirable by consumers in this particular food category (Bryhni et al., 2002). Therefore, these results suggest that lactic acid bacteria strains isolated from meat sources could be used as potential starter cultures to improve the sensorial properties of traditional fermented dry sausages (Quijada et al., 2018).

### 3.8 Challenge test against *Listeria monocytogenes*

**Fig. 5:**
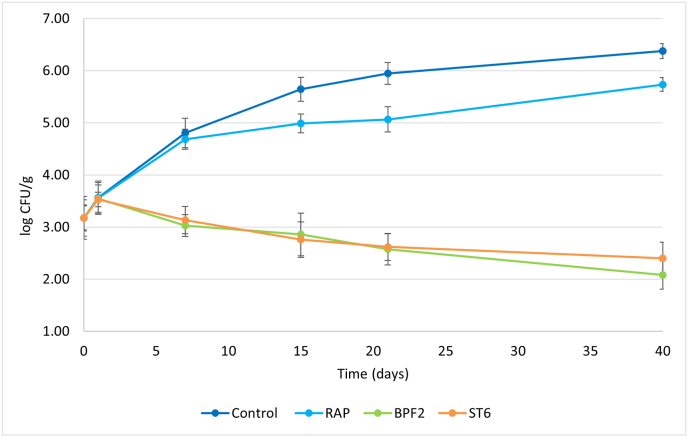
*L. monocytogenes* growth kinetics in the challenge test performed on Spanish fermented sausages.

With the aim to evaluate the effective bioprotective activity of the different starter cultures, fermented sausages were prepared under the same conditions previously adopted, but the meat batter was inoculated with *L. monocytogenes* DSM 112142 at a concentration of about 3.2 log CFU/g. During fermentation and ripening, the concentration of the pathogen was monitored, and the results are reported in Fig. 5.

In the control without starter cultures *L. monocytogenes* constantly grew and reached a final concentration of 6.4 log CFU/g. In the RAP samples a similar behavior was observed, even if after 7 days the concentration was lower, and the final value was 5.7 log CFU/g. A different situation characterised the samples inoculated with BPF2 and ST6. In fact, after a small initial increase (counts of approx. 3.5 log CFU/g after 1 day), the viable counts of *L. monocytogenes* progressively decreased and reached a final concentration of 2.1 log CFU/g and 2.4 log CFU/g in the presence of BPF2 and ST6, respectively.

*L. monocytogenes* is one of the main safety concerns in the production of Mediterranean salamis (Meloni, 2015). In fact, the thermal profiles used during fermentation and ripening and the reduction of NaCl initial concentration, pursued in the last decades, can favor the growth and survival of this pathogen (Barcenilla et al., 2022). In addition, the trend to reduce or eliminate the use of nitrate and nitrites can represent a further risk for listeria growth. Different solutions have been proposed to counterbalance this increased risk. Among them, the use of bioprotective cultures plays a crucial role to inhibit listeria growth or favor its death (Holck et al., 2017; Tabanelli et al., 2022). The new strains used in these trials demonstrated a relevant inhibiting activity against *L. monocytogenes*, not only if compared to the spontaneously fermented control, but also if compared with the commercial starter culture RAP.

*P. acidilactici* strains and their pediocins have been extensively documented as effective biocontrol agents against *L. monocytogenes* in meat products (Choyam et al., 2019; Khorshidian et al., 2021). The use of bacteriocin-producing strains and pediocins offers numerous advantages for the control of *Listeria* in meat products. *P. acidilactici* strains typically grow and survive under the demanding conditions of meat processing, including low pH levels and high salt concentrations. Moreover, pediocins are known to possess high heat stability, enabling them to withstand processing conditions encountered in the meat industry (Khorshidian et al., 2021). In accordance with our findings, other authors have also recently applied a pediocin PA-1-producing strain of *P. acidilactici* for the control of *L. monocytogenes* in fermented meat sausages, with significant reductions in counts of the pathogen (Komora et al., 2021). In addition, the same authors reported the *in situ* biosynthesized pediocin PA-1 in a similar fermented meat model. This potential capability to produce pediocin during the production of dry fermented sausage has also been previously documented by other authors (Foegeding et al., 1992). Therefore, this fact may be responsible of antimicrobial activity against *L. monocytogenes* described in present work.

Similarly, the anti-listerial activity observed in batches treated with *Lpb. Paraplantarum* strain BPF2 may be directly related to its ability to produce leucocin K. Leucocins are bacteriocins produced by certain bacterial strains, mainly *Leuconostoc* spp., that exhibit strong antimicrobial activity against *Listeria* and other related pathogens (Shi et al., 2016; Chen et al., 2018). Given that *Leuconostoc* is a genus commonly associated with the spoilage of meat products (Borch et al., 1996), there is scarce literature on the application of leucocins for controlling *Listeria* in food models. However, certain bacteriocinogenic strains of *Leuconostoc carnosum* have been reported by some authors as a bioprotective culture against *L. monocytogenes* in vacuum-packaged meat sausages (Budde et al., 2003). In our study, BPF2 strain exhibits a good sensory profile and is well adapted to fermentative processes, suggesting its potential use as a starter or bioprotective culture. This could provide new insights into the potential effects of leucocin K in fermented meat products. Finally, both pediocins and leucocins demonstrate a broad spectrum of antimicrobial activity, not only against *Listeria* but also against other common Gram-positive foodborne bacteria (Parente et al., 1996; Ghosh et al., 2019). Therefore, the potential use of these bacteriocinogenic strains could be further explored in controlling other pathogens and spoilage microorganisms.

## 4 Conclusions

The technological parameters of the fermented sausages obtained through a spontaneous fermentation or with the addition of different starter cultures presented differences during ripening. In particular, the weight loss was lower in the control samples, while the pH decrease was more relevant in the sausages RAP and BPF2, characterised by the prevalence of *Lactiplantibacillus* among LAB. The metagenomic data underlined that *Lpb. Paraplantarum* BPF2 had good ability to colonize the matrix, while *P. acidilactici* ST6 did not show a good fermentative performance, resulting in a lower percentage if compared with the percentages of the other LAB used as starter cultures.

The use of the starter cultures reduced the BA concentration observed in the sausages obtained with spontaneous fermentation. In addition, the sausages presented interesting differences in relation to the volatile profile. In particular, the use of the two bacteriocinogenic strains (*Lpb. Paraplantarum* BPF2 and *P. acidilactici* ST6) reduced the level of rancidity of the sausages as demonstrated by the volatilome profile and sensory analysis.

The use of the two autochthonous strains, selected based on their technological and bioprotective features against *L. monocytogenes*, proved to be extremely effective in counteracting the presence of this pathogen in salami during the challenge test, confirming their ability to produce bacteriocins also in a real system. In fact, the results obtained were highly ameliorative when compared to both the spontaneously fermented sample (control) and the sausage produced using the commercial starter culture (RAP), although the final occurrence of *Pediococcus* was found not to be predominant.

These aspects can make the use of these new strains interesting for an enhancement of the safety of traditional products and for their sensory differentiation, increasing the possibility for tailor made fermentation aimed to improve the recognizability and the peculiar traits of fermented sausages.

## CRediT authorship contribution statement

**J. David García-López:** Investigation, Visualization. **Federica Barbieri:** Investigation, Writing – original draft. **Alberto Baños:** Investigation, Methodology. **Jose Manuel Garcia Madero:** Conceptualization. **Fausto Gardini:** Writing – review & editing. **Chiara Montanari:** Investigation, Methodology. **Giulia Tabanelli:** Supervision, Conceptualization.

## Declaration of competing interest

The authors declare that the research was conducted in the absence of any commercial or financial relationships that could be construed as a potential conflict of interest.

## Data Availability

All data are available in the manuscript
